# The Canteen Bill in Congress

**Published:** 1906-02

**Authors:** 


### The Canteen Bill in Congress.

A bill has been introduced into the Lower House of Congress, by Representative Morrell, to permit the restoration of the canteen feature in the post exchanges of the army. This bill is supported by all the true friends of the soldier in and out of the army, but Mr. Hull, chairman of the House Committee on Military Affairs, is quoted as opposing any legislation of this nature during the present session. That is to say, if Mr. Hull has his way, the pimps, the rumsellers, the gamblers, and the prostitutes are to be given another year in which to prey upon the bodies and souls of the enlisted men, while the friends of the soldier, who, recognizing the futility of blue laws, would remove the temptation to degrading vice by giving opportunity for innocent recreation, are to be restrained by the firm hand of the law.

The reason alleged by Mr. Hull for inaction this year is the same as that expressed a year ago. He would wait, he says, until the present order of things has had a full trial! We wonder how long it will be before the divekeepers and their dupes and allies of the W. C. T. U. will admit that the present system has had a fair trial! If the roll of infraction of discipline, desertion, insanity, and death, directly traceable to the abolition of the canteen, is not sufficiently large in five years, will it be any more convincing to the illogical ladies who have so long terrorized Congress, in ten years —or fifteen?

A recent dispatch to the *New York Times* from Chicago gives some details of a grand jury investigation of saloon dives, gambling resorts, and other disorderly places in the town of Highwood, near Fort Sheridan. The evidence introduced by Capt. M. E. Saville and other officers of the fort alleged the following state of affairs existing as a direct result of the abolition of the canteen: "Twenty-five violent deaths in eighteen months as a result of soldiers frequenting the dives. Carloads of uniforms and other equipment stolen from the fort by soldiers and sold to divekeepers for liquor. Connivance at the desertion of soldiers by divekeepers. Every saloon, with one exception, is a gambling house, and all the games are 'crooked.'" It was also alleged before the jury that certain politicians and several wealthy individuals are in a ring to prevent the government from interfering with the "prosperity" of the village. These ringsters have also the powerful support of the Woman's Christian Temperance Union. Against this array of facts the opponents of the canteen have never, so far as we have been able to learn, offered anything tangible in proof of the alleged evils of selling beer in the post exchanges. Their arguments are based on pure assumption. It is a sin to drink beer, *ergo,* the sale of beer and light wines, even under strict limitation as to quantity, is an offense against morality. Let the men drink all the whisky they will, while on leave, but to remove the temptation to drunkenness by sanctioning moderate indulgence on the government reservation—never! Occasionally, it is true, a rara avis is found, even in the army, who sings a weak denial, in the face of the adverse testimony of an overwhelming majority of the officers and men, that drunkenness is any more common among the enlisted men today than it was in the canteen times. But such piping sounds faint through the virile roar of those who know the truth and speak it.

The real difficulty in the way of restoring the canteen at this session of Congress, according to the Washington correspondents of the *Globe* and other newspapers, is the fact that members of the House who want to succeed themselves must secure renomination and re-election within less than a year. Few of the representatives have the courage to inject deliberately the temperance question into their campaigns. In many parts of the country a vote for the canteen by a member of the House would be the signal for a war upon him by the whisky men and the W. C. T. U., and many a seat would be lost in consequence. Hence the reluctance shown in taking up the canteen question again.

The Association of Military Surgeons of the United States has memorialized Congress in favor of Mr. Morrell's bill, and the same also has the support of all the officers of the army, including the generals commanding the various departments and the chaplains, and all others, in and out of the service, who are in position to see the fearful evils entailed by the present system. The members of the House freely admit in private conversation the validity of the arguments in favor of the canteen, and we can not but think that there will be found a sufficient number of them, guided by conscience and with the courage of their convictions, to repeal the iniquitous anti-canteen law and restore to the enlisted man the protection against vice and the miseries which indulgence in it entails, of the beneficent post exchange with the harmless canteen feature restored. Surely common sense and morality must finally prevail against blind fanaticism and the crafty play upon it of the rumsellers and divekeepers.—*New York Medical Record.*

